# Proactive community case management in Senegal 2014–2016: a case study in maximizing the impact of community case management of malaria

**DOI:** 10.1186/s12936-020-03238-0

**Published:** 2020-04-25

**Authors:** Seynabou Gaye, Janelle Kibler, Jean Louis Ndiaye, Mame Birame Diouf, Annē Linn, Alioune Badara Gueye, Fatou Ba Fall, Médoune Ndiop, Ibrahima Diallo, Moustapha Cisse, Mady Ba, Julie Thwing

**Affiliations:** 1 Senegal National Malaria Control Programme, Dakar, Senegal; 2 United States Peace Corps, Dakar, Senegal; 3 Laboratoire de Parasitologie et Mycologie Médicale, Université Cheikh Anta Diop, Dakar, Senegal; 4 United States Agency for International Development, Dakar, Senegal; 5 U.S. President’s Malaria Initiative, Dakar, Senegal; 6 United States Agency for International Development, Washington, DC USA; 7 U.S. President’s Malaria Initiative, Washington, DC USA; 8 Division of Parasitic Diseases and Malaria, Malaria Branch, Center for Global Health, Centers for Disease Control and Prevention (CDC) Atlanta, Atlanta, GA USA

**Keywords:** Malaria, Diagnosis, Treatment, Community case management, Proactive

## Abstract

The Senegal National Malaria Control Programme (NMCP) introduced home-based malaria management for all ages, with diagnosis by rapid diagnostic test (RDT) and treatment with artemisinin-based combination therapy (ACT) in 2008, expanding to over 2000 villages nationwide by 2014. With *prise en charge à domicile* (PECADOM), community health workers (CHWs) were available for community members to seek care, but did not actively visit households to find cases. A trial of a proactive model (PECADOM Plus), in which CHWs visited all households in their village weekly during transmission season to identify fever cases and offer case management, in addition to availability during the week for home-based management, found that CHWs detected and treated more cases in intervention villages, while the number of cases detected weekly decreased over the transmission season. The NMCP scaled PECADOM Plus to three districts in 2014 (132 villages), to a total of six districts in 2015 (246 villages), and to a total of 16 districts in 2016 (708 villages). A narrative case study with programmatic results is presented. During active sweeps over approximately 20 weeks, CHWs tested a mean of 77 patients per CHW in 2014, 89 patients per CHW in 2015, and 90 patients per CHW in 2016, and diagnosed a mean of 61, 61 and 43 patients with malaria per CHW in 2014, 2015 and 2016, respectively. The number of patients who sought care between sweeps increased, with a 104% increase in the number of RDTs performed and a 77% increase in the number of positive tests and patients treated with ACT during passive case detection. While the number of CHWs increased 7%, the number of patients receiving an RDT increased by 307% and the number of malaria cases detected and treated by CHWs increased 274%, from the year prior to PECADOM Plus introduction to its first year of implementation. Based on these results, approximately 700 additional CHWs in 24 new districts were added in 2017. This case study describes the process, results and lessons learned from Senegal’s implementation of PECADOM Plus, as well as guidance for other programmes considering introduction of this innovative strategy.

## Background

The introduction of rapid diagnostic tests (RDTs) and artemisinin-based combination therapy (ACT) dramatically expanded access to prompt and effective case management of malaria in sub-Saharan Africa. However, inhabitants of remote, rural areas still face substantial challenges in accessing care, including geographical, educational and financial barriers, resulting in reduced effective malaria case management [1]. In rural Senegal prior to the introduction of community case management for malaria, roughly 90% of fever cases were first treated at home, and only 20% of cases received formal care [2]. Introduction of community case management has been an attempt to address these obstacles. In many scenarios, community case management has improved outcomes for childhood malaria, diarrhoea and pneumonia, and for all-cause child mortality [3–5].

Senegal was one of the first countries to bring diagnosis with RDTs and treatment with ACT to scale at the community level [6]. The country has a long-standing system of informal community-based health huts (*cases de santé)*, begun in 1977, supported by a network of non-governmental organizations and staffed by several cadres of community health volunteers. ACT was introduced at health facility level in 2006, followed by RDTs in 2007 [6]. In health huts, ACT and RDTs were introduced in 2008. To further address barriers to care, the Senegal National Malaria Control Programme (NMCP) introduced home-based management of malaria for individuals of all ages, known by its French acronym of PECADOM (*prise en charge à domicile*). Selected villages at least 5 km from a health facility and not served by a health hut chose a community member to be trained on case management of fever with RDTs and ACT. This cadre of community health worker (CHW) is known as a DSDOM, or *dispensateur de soins à domicile*. After a 20-village pilot in 2008 [7], the programme was scaled up to 408 villages in seven of Senegal’s 14 regions in 2009 and 861 villages in nine regions in 2010 [8]. Since then, it has been scaled up to over 2000 villages in 10 (of the 14) regions with higher transmission considered to be remote (≥ 5 km from a health facility) or with other difficulties in access to health facilities. In 2012, after extensive discussions with other divisions of the Ministry of Health and Social Welfare and with financial and technical partners, the NMCP and partners piloted the integration of management of diarrhoea and pneumonia for children under 5years old into the programme, and trained existing CHWs over the next 3 years to diagnose and treat diarrhoea and pneumonia among children under 5 years [9].

Despite improvements in access to care [8], limits were noted in the PECADOM strategy, especially due to low utilization. Even with the presence of trained health volunteers in communities, care seeking was sub-optimal. Inadequate supervision and frequent stock-outs were additional challenges noted during the first 4 years of PECADOM [10, 11].

The Kedougou region of Senegal provides an excellent example of the challenges facing remote, rural areas. Situated in the southeast of Senegal, with a regional capital 685 km from the capital city of Dakar, bordered by Mali to the west and Guinea to the south, Kedougou region had a population of 151,715 in 2013, with nine inhabitants per sq km [12]. Most inhabitants are subsistence farmers, though artisanal gold mining provides a livelihood for many. While Senegal reported a national annual malaria incidence in 2016 of 24 per 1000 inhabitants, reported annual malaria incidence was 300 in Kedougou region [13]. In 2010, Kedougou region had the highest all-cause child mortality and malaria parasite prevalence in Senegal [14]. In 2012 and 2013, the health district of Saraya (in Kedougou), in partnership with the US Peace Corps, the US President’s Malaria Initiative and the NMCP, piloted a proactive case management component to the PECADOM programme during the approximately 20 weeks of malaria transmission season. In the proactive model of PECADOM, known in Senegal as PECADOM Plus, CHWs and their communities chose 1 day each week to conduct household visits, or ‘sweeps’. Some community members (usually women) were trained to identify symptoms of malaria and assist CHWs in identifying residents in need of care. During the sweeps, CHWs attempted to visit every household in the community to identify residents with fever or history of fever during the previous 2 days. Every resident identified with fever or history of fever was tested by RDT, and those with positive RDTs received ACT.

In 2012, during the high transmission season (from August to November), PECADOM Plus was piloted in the five villages of the catchment zone of one health post; 563 symptomatic people received RDTs during active sweeps, of which 404 were positive (71.7%), and all positives were treated with ACT. While sweeps started in five villages, unfortunately, shortages of RDTs and ACT shortly after sweeps began necessitated limiting weekly active sweeps to a single particularly remote village for the majority of the transmission season. Adequate supplies of RDTs and ACT were secured shortly before the end of transmission season to carry out an end of season sweep in all the villages. During the end of season sweep, the prevalence of symptomatic, RDT-confirmed malaria cases was six times higher in the neighbouring villages in which a CHW was present but not conducting active sweeps than in the village in which sweeps had occurred weekly [15].

The follow-up 2013 trial included 14 intervention and 15 comparison communities (one CHW per community) in which sweeps were conducted at baseline, midline and endline, and in which a CHW was present for consultation through the standard passive PECADOM model, but not conducting active sweeps [16]. Mean population of each village was approximately 300, and households tended to be large (mean size 10 residents [14]) and relatively clustered. During the 21 weeks of the trial, CHWs in the 14 intervention communities performed 1036 RDTs during active sweeps, of which 62.4% were positive and treated with ACT. The prevalence of symptomatic, parasitologically confirmed malaria infection during the weekly sweeps was comparable at baseline in intervention (1.88%) and comparison (1.58%) villages, but at endline, during the 21st week of sweeps, this prevalence was 16-fold higher in comparison than intervention villages. In addition, the number of RDTs performed by CHWs between sweeps (indicative of care seeking) more than doubled in the intervention villages.

## Setting of PECADOM Plus scale-up

**Fig. 1:**
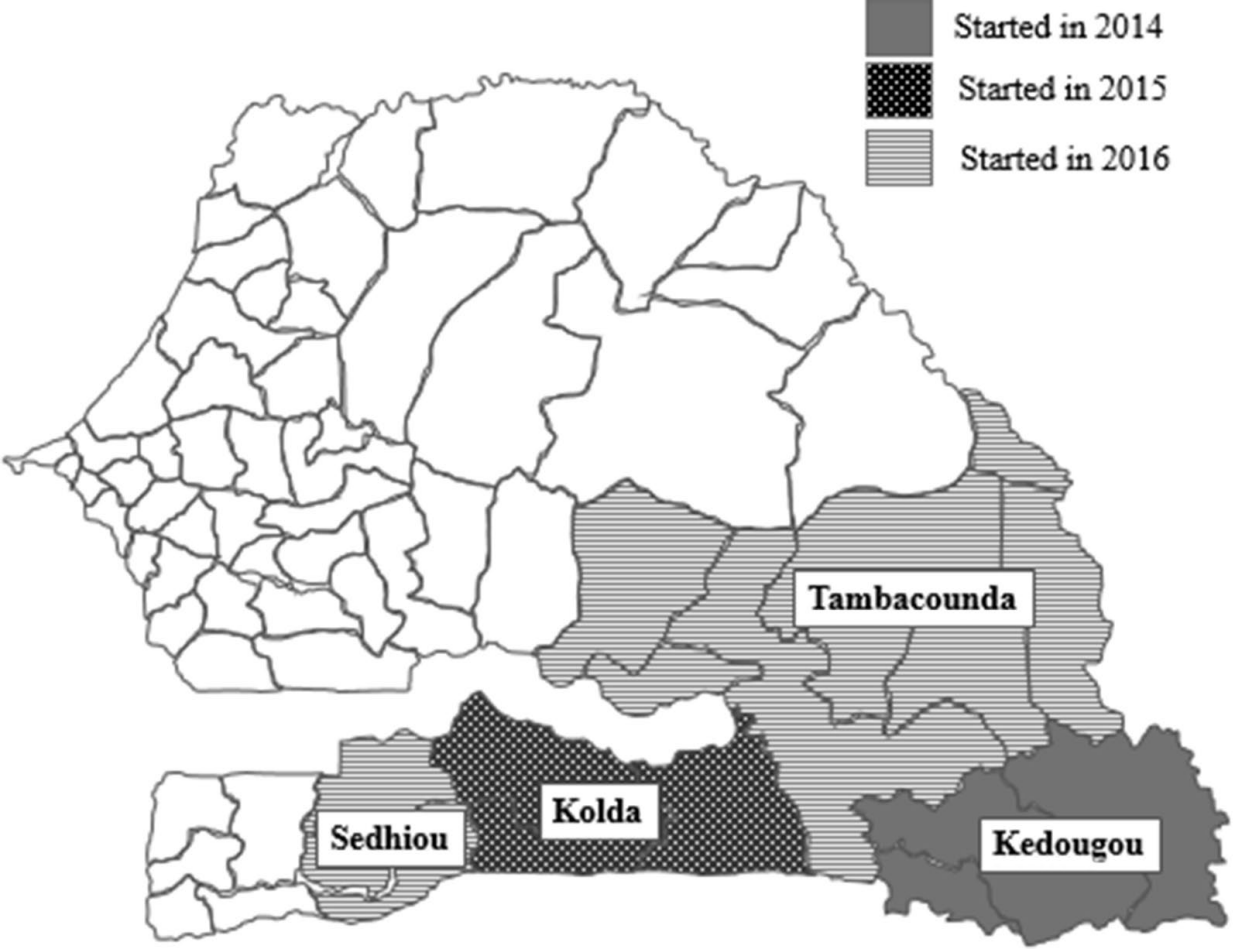
Senegal PECADOM Plus scale-up by region and year, 2014 to 2016

**Fig. 2:**
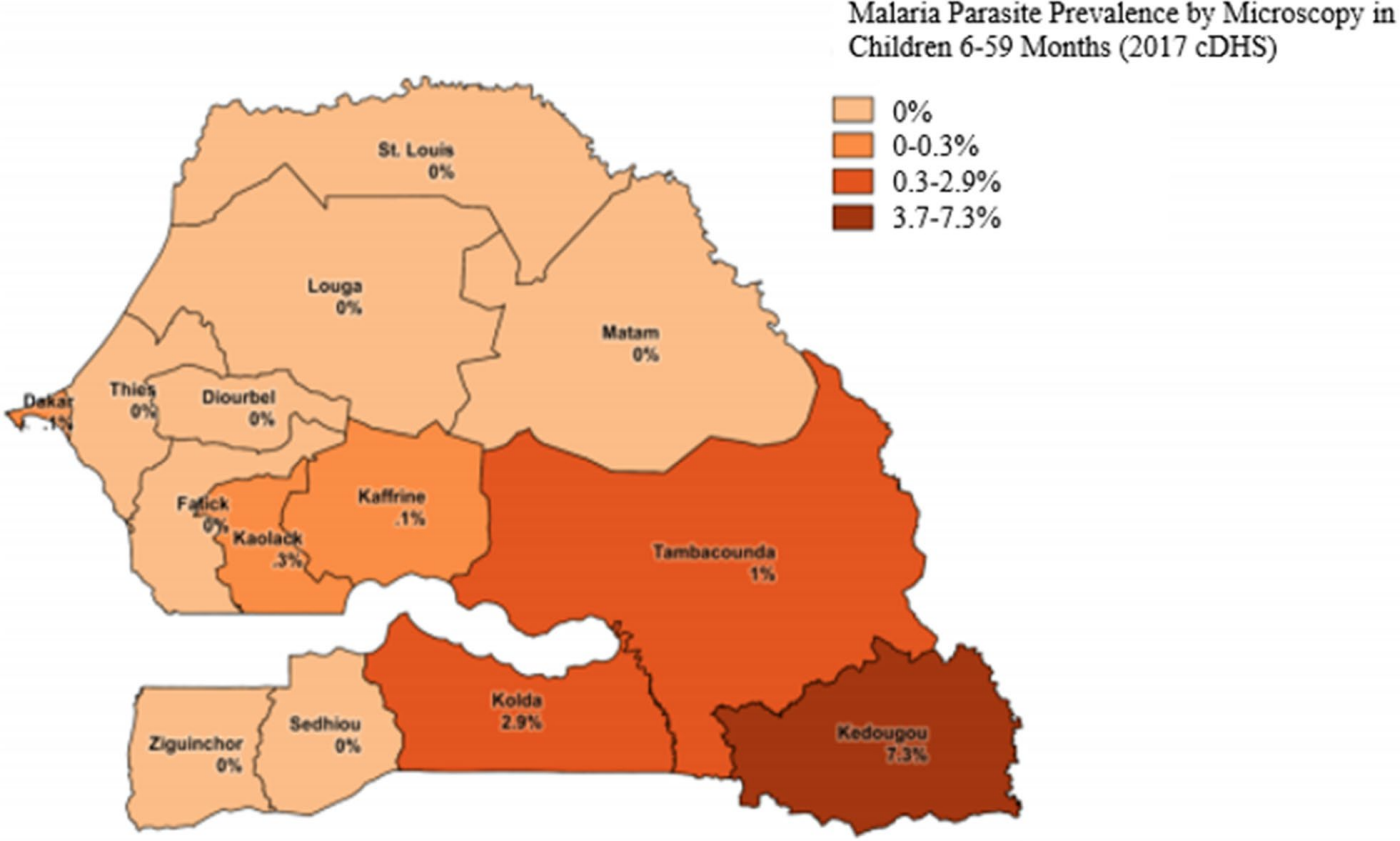
Parasite prevalence by region in Senegal, 2017

With these results, the NMCP adopted PECADOM Plus as a strategy, and introduced it to all the PECADOM villages in the region of Kedougou (132 villages) in 2014, adding all the PECADOM villages in the neighbouring similarly high transmission Kolda region in 2015 (for a total of 246 villages), and all the PECADOM villages in the remaining high transmission regions of Tambacounda and Sedhiou in 2016 (for a total of 708 villages) (Fig. 1). While the NMCP considers Tambacounda and Sedhiou to be part of the highest transmission zone, along with Kedougou and Kolda, transmission is lower in Tambacounda and substantially lower in Sedhiou (Fig. 2). Diagnosis and treatment of diarrhoea and pneumonia among children under5 years was incorporated into the active sweeps in 2014. Other interventions also took place during this period: long lasting insecticidal nets (LLINs) were distributed in Kedougou in July 2013, in Kolda in November 2013, and in Tambacounda and Sedhiou in May 2014, and again in all four regions in March 2016. The Ministry of Health adopted a policy of free care for children under 5 years in 2014. Seasonal malaria chemoprevention (SMC), which in Senegal targets children aged 3 to 120 months, was fully implemented in all four regions starting in 2014: with four rounds in Kedougou (July–October) and three rounds in Kolda, Tambacounda, and Sedhiou (August-October).

## Methods

This case study describes the introduction of the PECADOM Plus model in Senegal as implemented by the Senegal NMCP and regional and district health teams. The approach followed by the NMCP is detailed, programmatic results from the first 3 years of scale-up (2014–2016) are presented, and lessons learned and recommendations for future study and implementation are discussed.

Programmatically collected data from the first 3years of the scale-up, from both the active sweeps and the regular passive case detection and case management activity of the CHWs are presented, with a focus on the malaria data. Community supervisors collected data from DSDOMs weekly, including whether the DSDOMs had performed the household visits for that week, the number of people seen for fever, cough, and diarrhoea, the number of RDTs performed, the number of RDTs positive, the number of people treated for malaria, as well as for diarrhoea and pneumonia, and the numbers referred (for severity, infancy, pregnancy, and stock-out). DSDOMs also reported these figures for patients seen between weekly household visits, when patients sought care (passive case detection). These were aggregated monthly and reported to the district health officials and to the NMCP. Simple descriptive statistics were used to present totals of people tested and diagnosed both passively and actively, as well as those referred. In the context of programmatic scale-up rather than a study setting, detailed information on village population and location, distance from the health post, household size, and weekly household visit coverage were not reported. Cases diagnosed at health posts are not reported by village, thus differences in cases seen at the health post level were not measured and are not reported.

## PECADOM plus model and implementation

Successful implementation of this strategy required a comprehensive approach to training, supervision, supply chain, and communication, and included the following activities, starting with training, describing the household visits, and then describing supporting elements necessary to support the program:

1. Orientation of the regional health management teams (RHMT) and district health management teams (DHMT) in a regional workshop to share the guidelines of the strategy. This orientation equipped the management teams to organize their personnel at the level of the district for implementation and monitoring.
2. Training of health post chief nurses (HPCN): following the training of regional and district level teams, the DHMT, supported by representatives of the RHMT and a representative of the central level, organized workshops in their districts to train the HPCN who supervised the CHWs on the guidelines and the implementation of PECADOM Plus.
3. Selection of community supervisors: usually experienced CHWs, these individuals liaised between the CHWs and health posts, provided supervision and facilitated both reporting and supply chain management.
4. Training of community supervisors and CHWs at the district level: all of the CHWs and community supervisors in each district, the key players in the implementation, were trained by the DHMT, supported by a member of the RHMT and a representative of the NMCP. As CHWs had already received integrated community case management (iCCM) training, the 5 day classroom training included modules on the PECADOM Plus approach, refresher training on malaria, diarrhoea, and acute respiratory illness (ARI), and monitoring and evaluation.
5. Practical training of CHWs at health posts: after the classroom-based training at the district level, CHWs participated in 15 days of practical training at the health post supervising them. This internship allowed CHWs to practice RDT performance and breath counting for pneumonia diagnosis, as well as correct treatment. (Of note, CHWs not participating in the proactive component receive the same training.)
6. Supplies for CHWs: after the practicum, the CHWs were provided with equipment (timer, thermometer, bag, cap, vest, T-shirt, case register, badge) and supplies (RDTs, ACT, oral rehydration solution (ORS), zinc, dispersible amoxicillin) to enable them to carry out the planned sweeps.
7. Communication/awareness: HPCN generally visit the villages in their catchment zones every 1 to 3 months, and have established relationships with community leaders. Each HPCN met with community leaders to inform them about the work of the CHWs and the weekly sweeps, and to obtain their support. CHWs educated community members on the services provided through PECADOM Plus, prevention, early treatment seeking, and adherence to treatment. The CHWs selected community members (usually women) in each cluster of households, whom they trained on malaria symptoms and danger signs, and who assisted the CHWs in identifying people needing care.
8. Weekly household sweeps: once every week during the malaria transmission season, the CHWs went door to door to every household in their respective villages to detect, test and treat or refer suspected cases of malaria, diarrhoea or ARI. Any suspected case of malaria (determined by a fever or history of fever in the last 48 h) received an RDT. A thermometer was used to measure the temperature. All cases of uncomplicated malaria without indication for referral were treated with ACT. Patients with negative RDT, temperature greater than 39.5 °C, signs of severe disease, children under 2 months, pregnant women in the first trimester, or in case of stock-out, were referred to the closest health facility. Children diagnosed with diarrhoea were treated with ORS and zinc and those diagnosed with pneumonia were treated with amoxicillin. If the CHW had a stock-out of tests or any treatment, weekly sweeps continued, and residents were referred to the health post if the RDT or medication needed was not on hand.
9. Remuneration: both CHWs and community supervisors received a stipend for each day of work ($5 for CHWs and $10 for community supervisors), similar to what was paid for similar level of effort for a day of work during public health campaigns. They also received funds for transportation to coordination meetings and telephone credit to facilitate communication.
10. Supervision: supervision was planned to be conducted every week by the community supervisor, every month by the HPCN, and quarterly by the DHMT. Community supervisors were requested to visit each CHW weekly, or in areas where larger distances separate their CHWs, every 2 weeks, observing their weekly sweeps, giving feedback, recording summary data, and making sure they had supplies. Community supervisors often brought supplies to CHWS.
11. Supply chain: needs for RDTs and ACT were estimated for PECADOM Plus villages prior to the transmission season, and the NMCP worked to assure that districts ordered and received sufficient quantities of RDTs and ACT for the community level for the transmission season. Other sections of the Ministry of Health and partners were responsible for medications to treat pneumonia and diarrhoea. Community supervisors mentored CHWs in monitoring their supplies and often facilitated resupplying CHWs. Supplies were received from the supervising health post.
12. Coordination: monthly coordination meetings included the CHWs, community supervisors, and HPCN. At the district level, the district medical officer (DMO) met with the DHMT and the HPCNs monthly. At these meetings, the status of the implementation, constraints and difficulties encountered, and proposed solutions were discussed. The NMCP PECADOM focal point participated in these meetings by remote conferencing, and was responsible for reporting to the NMCP Coordinator any constraints noted in the implementation and proposed solutions.
13. Monitoring and evaluation: monitoring was done at three levels: by community supervisors, HCPN and DHMT. An evaluation meeting was held after the malaria transmission season to share experiences among the various stakeholders. During these workshops, districts presented the results of the PECADOM Plus programme, analysed the implementation, and identified the strengths, weaknesses, lessons learned, and recommendations. In addition to these workshops, the results of PECADOM Plus programme were annually published in the NMCP bulletin and have been disseminated internationally at scientific meetings [17, 18].
14. Integration of development partners and non-governmental organizations (NGOs): while led by the NMCP, RHMT and DHMT, PECADOM Plus benefitted from the involvement and support of development and NGO partners. In the early scale-up of PECADOM Plus in Senegal, Peace Corps volunteers played integral roles in training, supervision and mentoring of CHWs, facilitating communication and supply chain, and participating in the monitoring and evaluation component.

## Results

### Active community sweeps

2014 in Kedougou: during the 20 weeks of the intervention, 132 CHWs saw 11,844 patients during sweeps (mean 5 patients per sweep), of which 84% had fever, 9% had diarrhoea, and 5% had cough. RDT positivity rate was 80% (8071/10,141) (Table 1). Treatment with ACT was given in 98.6% of RDT positive cases (29% of whom were < 5 years); 1.0% had signs of severity and 0.4% of the cases were referred because of drug stock-outs. Almost all diarrhoea cases (1031/1035) were either appropriately treated by the CHWs with ORS and zinc or referred (3% referred for ORS or zinc stock-out), as was the case for pneumonia (158 cases), though 40% of pneumonia cases had to be referred for stock-out of antibiotics. In total, 2317 patients were referred for care at health posts: 90% for a negative RDT, 4% for medication stock-out, 3% for severe malaria, 2% for pregnancy, and 1% for age under 2 months.

**Table 1:** Weekly sweep results for malaria case management by year and region during scale-up (2014–2016)

Region	Year	Number of villages (CHWs)^a^	Number of weekly sweeps reported completed (% of expected)	Number of fever cases	Positive RDTs/ RDTs performed (test positivity rate)	Mean number of positive RDTs per sweep
Kedougou	2014	132	2375 (90%)	9970	8071/10,141 (80%)	3.4
	2015	144	1898 (94%)	10,321	6095/9997 (61%)	3.2
	2016	165	3897 (99%)	15,382	9378/15,260 (61%)	2.4
Kolda	2015	102	1462 (87%)	12,517	8973/11,918 (75%)	6.1
	2016	105	2372 (99%)	17,176	10,384/16,823 (62%)	4.6
Sedhiou	2016	150	2400 (100%)	9177	1115/9100 (12%)	0.5
Tambacounda	2016	288	5009 (95%)	22,433	9677/22,420 (43%)	1.9

^a^One CHW per village

2015 in Kedougou and Kolda: for 21 weeks in Kedougou and 19 weeks in Kolda, 246 CHWs saw 27,621 patients during active sweeps (mean 8 patients per sweep), of which 83% had fever (96% of whom received an RDT), 5% had diarrhoea, and 12% had cough. RDT positivity rate was 68%. Of those diagnosed with malaria (27% of whom were < 5 years), 96.1% received ACT, while 2.2% were referred due to ACT stock-out and 1.3% were referred due to signs of severity. Among children diagnosed with pneumonia (n = 1768), 41% were referred for stock-out of amoxicillin, and among children diagnosed with diarrhoea (n = 1379), 57% were referred due to stock-out of ORS or zinc.

2016 in Kedougou, Kolda, Tambacounda, and Sedhiou: for 25 weeks in Kedougou, 22 weeks in Kolda, 19 weeks in Tambacounda, and 16 weeks in Sedhiou, 708 CHWs saw 64,168 patients during active sweeps (mean 6 patients per sweep), of which 77% had fever (99% of whom received an RDT), 9% had diarrhoea, and 13% had cough. RDT positivity rate was 48%. Of those diagnosed with malaria (21% of whom were < 5 years), 99% received ACT. The remaining 1% were referred for signs of severity. The majority of children diagnosed with diarrhoea or pneumonia had to be referred due to lack of availability of medication for diarrhoea and pneumonia.

Table 1 summarizes the results of the weekly sweeps by region and year for 2014–2016.

### PECADOM passively detected malaria cases

During the scale-up of PECADOM Plus, cases detected and treated during passive work by CHWs (the standard PECADOM model with care being sought by community members outside of weekly sweeps) also increased among CHWs who did proactive sweeps. From the year prior to introduction to the first year of implementation in each region (2013 to 2014 in Kedougou, 2014 to 2015 in Kolda, 2015 to 2016 in Sedhiou and Tambacounda), the number of RDTs performed by CHWs during passive work increased by 56% in Kedougou (from 4473 to 6959), 112% in Kolda (from 5298 to 11,254), 110% in Tambacounda (from 13,283 to 27,840), and 139% in Sedhiou (from 3490 to 8346). During the same period, the number of cases diagnosed by CHWs during passive work increased by 61% in Kedougou (from 3328 to 5363), 149% in Kolda (from 3126 to 7787), 54% in Tambacounda (from 7247 to 11,462), and 87% in Sedhiou (from 453 to 849) (Table 2). (The number of CHWs increased by 2% in Kedougou, decreased by 1% in Kolda, and increased by 12% and 10% in Tambacounda and Sedhiou, respectively.) While the largest increases were seen during the first year of implementation, the numbers of people receiving diagnostic tests and diagnoses of malaria in between sweeps continued to increase during years two and three in Kedougou and year two in Kolda.

**Table 2:** PECADOM passively detected malaria cases by region and year

Region	Phase	Year	Number of villages (CHWs)^a^	Fever cases	Positive RDTs/ RDTs performed (test positivity rate)	% of all cases detected by DSDOM^b^ that were detected during passive work
Kedougou	Pre (trial)	2013	129	4507	3328/4473 (74%)	All
	Year 1	2014	132	7206	5363/6959 (77%)	40%
	Year 2	2015	144	8078	5074/7633 (66%)	46%
	Year 3	2016	165	13,435	8981/13,974 (64%)	49%
Kolda	Pre	2014	104	5935	3126/5298 (59%)	All
	Year 1	2015	102	11,930	7787/11,254 (69%)	47%
	Year 2	2016	105	18,892	10,321/17,969 (57%)	50%
Tambacounda	Pre	2015	258	15,116	7247/13,283 (55%)	All
	Year 1	2016	288	27,750	11,162/27,840 (41%)	54%
Sedhiou	Pre	2015	136	3523	453/3490 (13%)	All
	Year 1	2016	150	8390	849/8346 (10%)	43%

^a^One CHW per village

^b^Actively and passively

### Total malaria diagnoses by CHWs

**Fig. 3:**
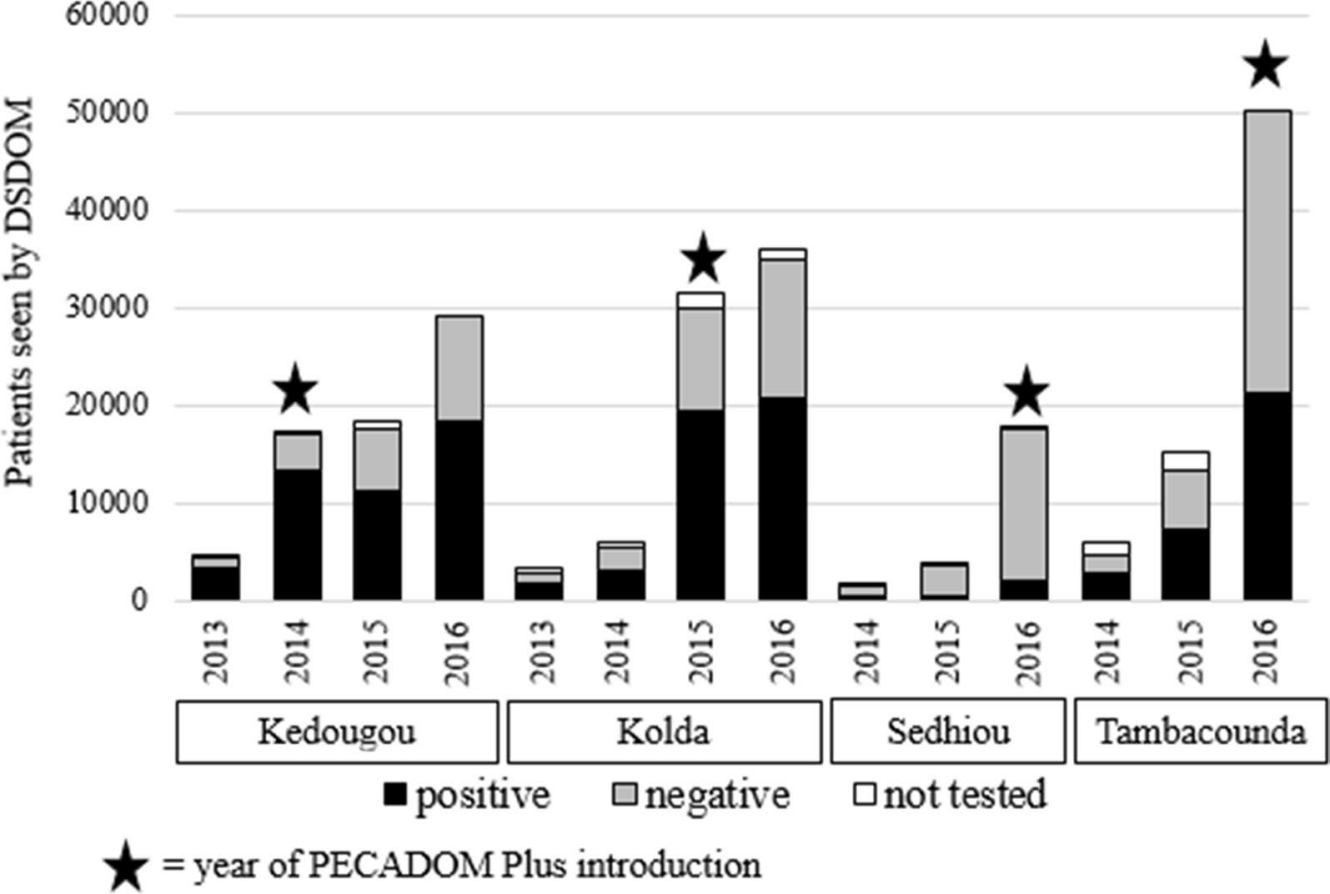
Total fever cases managed by DSDOM by year and region during introduction of PECADOM Plus, including both actively and passively detected cases, by test status

**Fig. 4:**
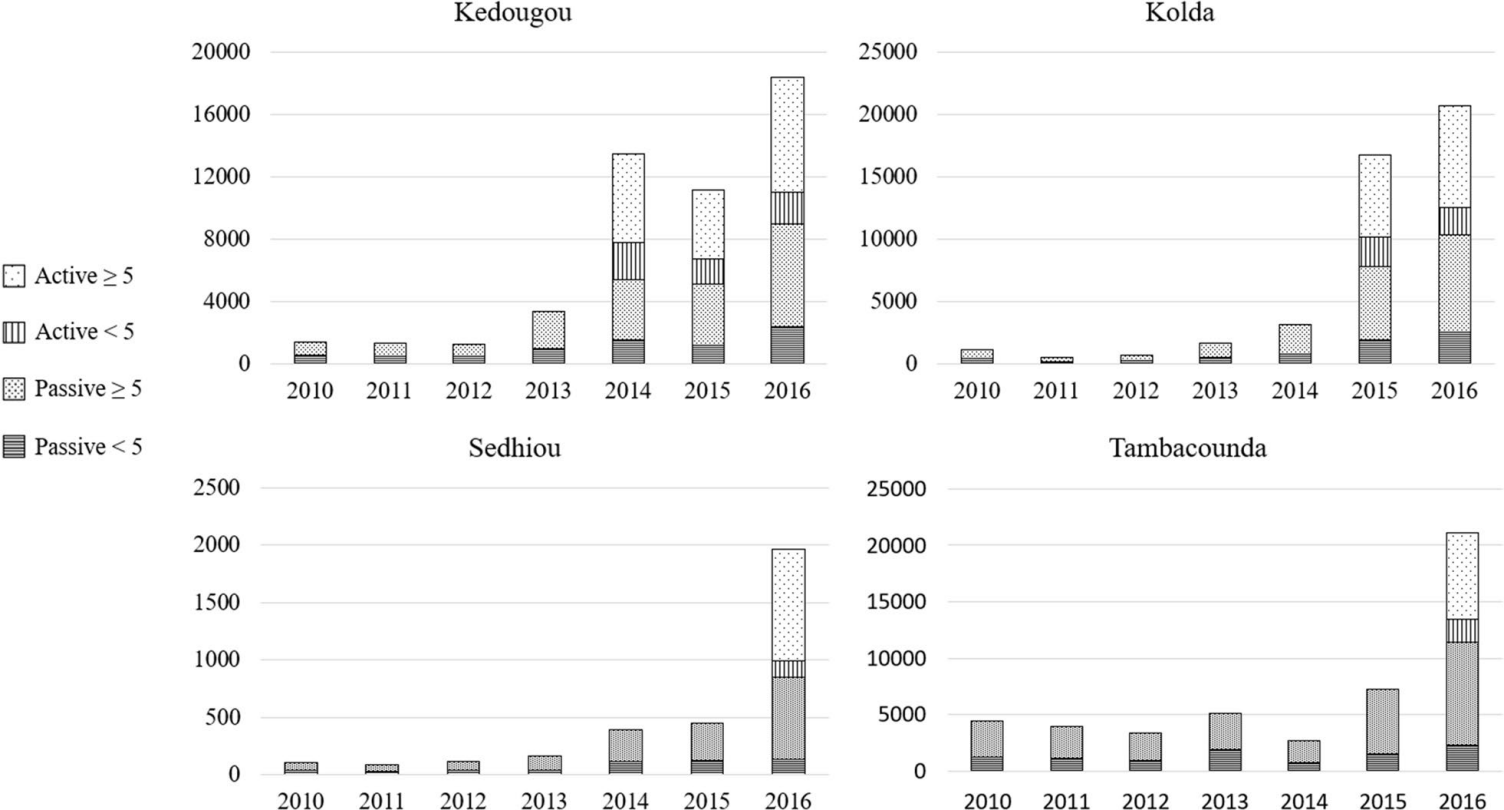
Malaria cases diagnosed by community health workers, by region, of passively and actively detected cases, among children < 5 years and residents ≥ years, 2010–2016

The increase in the total number of malaria cases detected (actively and passively) by CHWs from the year prior to implementation to the first year of implementation was 304% in Kedougou (2013 to 2014) and 436% in Kolda (2014 to 2015). Increases of 399% in the Sedhiou region and 232% in the Tambacounda region were seen from 2015 to 2016 (Figs. 3 and 4). The dramatic increase among cases diagnosed occurred among both children < 5 years and residents ≥ 5 years (Fig. 4). Overall, from the year prior to implementation to the first year of implementation, while the number of CHWs increased by 7% in the four regions, the mean number of malaria cases diagnosed per CHW during the year increased from 23 to 79, an increase of 252%. The most pronounced increase was in the Kolda region, where the number of CHWs stayed constant, but the mean number of malaria cases diagnosed per CHW increased from 30 to 164. Increases in numbers of cases diagnosed by CHWs were also noted from 2014 to 2015 in Tambacounda (172%) and Sedhiou (15%), the two regions that did not introduce PECADOM Plus until 2016, as efforts to improve the availability of malaria commodities at the community level for the PECADOM Plus regions were applied to other regions as well. In 2016, in the regions in which PECADOM Plus was implemented, 40% of all malaria cases reported were detected at the community level (at health huts and by CHWs), while in regions in which PECADOM Plus was not implemented, only 8% of reported malaria cases were detected at the community level.

### Learning lessons and addressing challenges

At the end of each season, the NMCP hosted an evaluation meeting in each region attended by regional and district health representatives and community stake holders, in which results from each district were presented, and feedback was solicited from stakeholders at all levels of the health system and community members. PECADOM Plus was positively received by communities, health post nurses and DHMTs. Nurses and district medical officers stated that they saw fewer cases of severe malaria. The implementation of weekly sweeps greatly increased the visibility of CHWs in their communities and increased community engagement. Populations with high poverty and low access, with relatively little exposure to modern medicine, were able to observe the efficacy of malaria diagnosis and treatment in their communities. In addition to case management, CHWs used the visits as opportunities to talk about use of insecticide-treated nets, intermittent treatment in pregnancy and SMC.

There was a dramatic increase in symptomatic, parasitologically-confirmed cases diagnosed by CHWs when the proactive component was introduced. The relative contributions of improved care seeking due to increased community engagement, RDT and ACT availability due to enhanced supply chain support, and enhanced supervision with improved reporting to the observed increases in reported cases are not possible to ascertain, though all likely played a role. However, it is likely that a large number of malaria cases had previously gone undetected and untreated in the community.

Much of this should have been seen in the passive model, however, without the added effort in supervision and supply chain management necessitated by the proactive community sweep component, as well as the higher visibility provided by the weekly visits, the potential of the passive model was not reached. As proactive detection of symptomatic cases led to detection and treatment of far more cases of malaria, it exposed weaknesses in the PECADOM programme that required resolution, most critically supply chain management, supervision, compensation of CHWs, coordination, and reporting.

Supply chain challenges were evident from the beginning of the first pilot in 2012, with insufficient RDTs and ACT to support the PECADOM Plus activities, even if stock-outs at health facilities were uncommon. It rapidly became apparent that CHWs had been routinely experiencing frequent and prolonged shortages of commodities, and emergency deliveries were required on numerous occasions as the NMCP and DHMT worked to develop more sustainable solutions, such as planned deliveries of commodities at the beginning of the malaria transmission season sufficient to support the community level. Partners such as Peace Corps played instrumental roles in assisting the community to alert district health officials to shortages and helping redeploy commodities. The improvement of the supply chain may also have been at least partially responsible for the apparent increase in care seeking at the community level. While the NMCP was able to address malaria commodities shortages, this was not the case for diarrhoea and pneumonia treatments during the time frame of this scale-up, as other partners were responsible for these. Except for diarrhoea treatments in Kedougou in 2014, approximately half of children diagnosed with diarrhoea or pneumonia had to be referred for treatment, which compromised the credibility of the integrated approach and discouraged the CHWs, some of whom reported that they stopped trying to identify cases of diarrhoea and pneumonia when they had no treatment to offer. Fortunately, the attention drawn to the shortage of non-malaria commodities at the community level later enabled resolution of these shortages.

Health post nurses had the responsibility to supervise CHWs, but the distances over difficult paths and their additional workload resulted in spotty supervision. The NMCP developed a cadre of community supervisors trained to directly supervise CHWs on a weekly basis, and to report to the health post nurse. These community supervisors were also instrumental in collecting data for programme monitoring and ensuring that the CHWs had sufficient commodities.

Previous community health projects did not financially compensate CHWs, based on the principle that the community should support them, however, the degree to which this happened was highly variable. Successful implementation of PECADOM Plus required compensation for CHWs. Artisanal gold mining is a common income-generating activity in this zone, and prior to the introduction of PECADOM Plus, some CHWs regularly left their villages to mine. Under PECADOM Plus, CHWs received a stipend (US$5) for each day that they performed an active sweep of the village (approximately 20 days per year), equivalent to the amount they typically received per day for work during public health campaigns (e.g. vaccination, deworming). While a few CHWs continued their mining activity away from the village on non-sweeps days, and occasionally missed a sweep, even the small stipend was sufficient to induce the majority of CHWs to stay in their communities and provide care. However, in cases in which the stipends were not paid in a timely manner, completion of sweeps sometimes suffered. To assure adequate supervision and participation in the monthly coordination meetings, resources were also needed for phone credit and transportation. Community supervisors also received a small stipend and funds for transportation and phone credit.

Effective coordination and monitoring required a great deal of time and support. Numerous partners assisted at every level of implementation. Regular coordination meetings at the health post and district levels were critical to rapid problem solving, and at the central level, NMCP staff provided support with problem resolution. Within health post catchment zones, frequent communication between community supervisors and CHWs was required, necessitating cell phone credit.

Obtaining quality data from the community level is a persistent challenge, but one not new to the active model. Many CHWs, with very basic levels of literacy, found filling out registers and summarizing data extremely challenging. While data collection forms were designed in such a way that data entry and collation involved making and counting checkmarks, data were still fraught with inconsistencies, and required time and attention from community supervisors and other partners to address. Other ongoing challenges included timely integration of community level data and reporting of sweep data, and assuring that the population covered by each CHW was small enough in numbers and geographic spread to allow a complete sweep each week.

## Discussion

By the end of 2016, PECADOM Plus had been introduced to 708 villages in Senegal’s four highest transmission regions for approximately 20 weeks during high transmission season. The number of cases diagnosed during active sweeps was slightly higher than the number diagnosed in passive case detection, though cases diagnosed passively doubled when the proactive component was introduced, resulting in increases of at least 300% in cases diagnosed and treated by CHWs in the four regions the year the active component was introduced. Scale-up under programmatic conditions did not permit weekly data collation and reporting, and it was not possible to analyse trends in the number of cases over the course of a transmission season in PECADOM Plus villages compared to health posts.

There had been concern that PECADOM Plus might have a perverse effect of decreasing care seeking, as people waited for the next sweep day. Instead, the number of cases diagnosed on non-sweep days increased dramatically as PECADOM Plus was introduced, suggesting that it may have functioned as a behaviour change intervention by increasing awareness of access to prompt diagnosis and treatment and demonstrating its effectiveness to remote communities that may have had limited positive interaction with the health care system [10].

During this period, the Senegal Ministry of Health was rolling out numerous strategies to improve access to care and care seeking, including insurance cooperatives, universal treatment free of charge for children under 5years, and adding health facilities in under-served areas. The NMCP improved the distribution of malaria commodities and encouraged providers to test all patients with febrile illness with an RDT; at the health facility level, an increase in all-cause consultations, tested and treated cases was also seen. In the four PECADOM Plus regions, from 2013–2016, the incidence of all-cause outpatient consultations increased by 47%, and the proportion of all outpatient consultations tested with RDTs increased from 23 to 31% [13, 19–21]. Simultaneously, other malaria control interventions were being intensified. From 2013–2014, and again in 2016, the four regions conducted mass distribution campaigns of long-lasting insecticidal nets, with the goal of covering every sleeping space. SMC started in these regions in 2014; children 3–120 months received a treatment course of sulfadoxine-pyrimethamine and amodiaquine once monthly during rainy season. Despite all these interventions, the reported incidence of confirmed cases increased by 29%, though the test positivity rate fell from 63 to 28% among children under 5 years and from 67 to 45% among the general population. Given the simultaneous introduction of other malaria control interventions and general health system strategies, ascertaining the impact of this programme alone on morbidity at the health facility level will be difficult. Modelling predicted that introduction of treatment with ACT in place of previously existing treatment would decrease parasite prevalence in a transmission setting such as southeastern Senegal [22], and it would be expected that a dramatic increase in the proportion of symptomatically infected individuals treated with an ACT would decrease parasite prevalence.

In particular, artemether-lumefantrine (the first line in these regions due to use of amodiaquine for SMC) is associated with a decreased duration of gametocytaemia compared to treatment with other anti-malarials, or to no treatment [23, 24]. While approximately 90% of children have been found to be gametocytaemic at presentation by molecular methods, and those with sub-microscopic gametocytaemia are infectious to mosquitoes, those with microscopically detectable gametocytaemia are more likely to infect mosquitoes [25]. Given that *Plasmodium falciparum* gametocyte maturation takes 8–10 days, detecting and treating symptomatic individuals with ACT on a weekly basis could potentially remove a substantial proportion of the infectious reservoir. In central Senegal, a dramatic decrease in malaria prevalence among children under 5years, from approximately 60 to 30%, was observed with the introduction of treatment with combination therapy, even prior to the introduction of insecticide-treated net distributions [26]. While evidence was equivocal when community case management of malaria was based on chloroquine during the years of waning chloroquine sensitivity [27], trials of community case management with ACT have been shown to have highly favourable results for the populations they serve [28, 29]. When paired with a proactive visit component, a larger number of infections are promptly detected and treated at the community level. In Mali, CHWs reached 35% of children within 24 h and 52% within 48 h, the proportion of children under five with fever who received treatment with an effective anti-malarial increased from 14.7 to 35.3%, and fever prevalence among children under five decreased from 39.7 to 22.6% [30, 31]. Results of the pilot study done in the Saraya district of Senegal [16], which ultimately led to the expansion of the PECADOM Plus programme, suggested that the prevalence of symptomatic malaria infection decreases sharply among populations in which weekly active case detection is implemented.

Data collected programmatically during the scale-up were not sufficiently granular to show this, but rather demonstrated that the number of cases diagnosed and treated at the community level increased dramatically with this approach. In Mali as well, care seeking increased both in health facilities and at the community level when proactive case detection was introduced [30]. Use of programmatically collected data to determine reduction of disease burden, when numerous other factors are changing simultaneously, is not possible, and the routinely conducted nationally representative cross-sectional surveys do not have sufficient granularity to evaluate the impact on parasite prevalence at the village level. The limitations in this case study are largely due to the challenges in reporting and obtaining quality data in this setting: it was not possible to verify the proportion of planned weekly visits that were completed, nor was it possible to verify the proportion of households visited weekly with a paper-based system that was manageable for the DSDOM. The population of PECADOM Plus villages is not reported, and health facility-based reporting does not differentiate between PECADOM Plus and other communities, hence reporting on cost per population covered or differentiating trends in incidence between PECADOM Plus and other communities is not possible. While the high test positivity rate in the setting of relatively low (< 10%) reported parasite prevalence may give pause; this was verified through independent supervision visits, and the cross-sectional surveys that measure parasite prevalence are conducted primarily during dry season when reported incidence is extremely low.

## Conclusion

In this setting, frequent household visits to proactively offer community case management to those with symptoms was feasible, well-accepted by communities, increased community engagement, increased the number of patients treated by CHWs, and might have decreased the proportion of a community infected with malaria. This approach should be considered where there is an existing community health infrastructure, though provisions must be made to reinforce the supply chain, supervision and monitoring structures to support effective community case management. A high degree of coordination is necessary between the central and district levels, at the district level, within a health facility catchment zone, and among partners, both at the central and operational levels, and DHMTs may need the support of non-governmental organizations to effectively support the community actors implementing the programme. Successful implementation of a programme that requires increased time commitment from CHWs also requires timely monetary compensation for the CHWs and supervisors, as well as support for transportation and coordination. Given the challenges in timely transmission of quality data and the increasing importance of strong community surveillance, mobile tools for minimally literate health workers should be developed, tested and deployed.

While the strategy was adopted in Senegal on the basis of a small trial conducted in the zone in which the strategy was adopted, there are many unanswered questions. It is not clear what the impact of the strategy is on parasite prevalence, or if it would be more effective if continued year round. While it was conducted in a zone of moderate highly seasonal transmission, the feasibility and effectiveness of the strategy in other transmission zones, beyond diagnosing and treating more cases at the community level, is unknown. While it might encourage prompt care seeking and reduce progression to severe disease, treatment of the small fraction of the population symptomatic each week may not be sufficient to decrease the transmission reservoir in higher transmission zones. In zones of lower transmission, it could be more cost effective and sustainable than strategies such as mass drug administration to further reduce transmission toward zero, but its effectiveness in further decreasing transmission in a low transmission zone is unknown. High quality, cluster randomized, controlled trials should be conducted to address these questions, and to better characterize cost and effectiveness of the proactive model. If adequate resources are given to data collection tools, particularly mobile data tools to collect data for each encounter, a powerful platform could be built for timely reporting and mapping of cases that if continued as transmission is driven down, could provide the basis for monitoring an elimination programme, while continuing to offer treatment for other major diseases of childhood.

While it is generally accepted that a high level of care and treatment at the community level and a system of community-based surveillance will be necessary to achieve malaria elimination, creating a functional, sustainable programme has proven challenging. The PECADOM Plus model may offer such an opportunity. It engages communities in regular detection and treatment of malaria, diarrhoea and pneumonia, and offers the opportunity to identify and treat a greater number of cases at the community level. In addition, the community engagement created provides a platform for integrating social and behavioural change communication, and monitoring of ITN availability and use. By increasing community engagement with and support to CHWs, it allows them to maximize their potential, and therefore the potential of the iCCM platform to improve the health of communities.

## Data Availability

The datasets used during the current study are available from the corresponding author on reasonable request.

## Abbreviations

ACT: Artemisinin-based combination therapy
ARI: Acute respiratory infection
CHW: Community health worker
DHMT: District health management team
DMO: District medical officer
DSDOM: Distributeur de soins a domicile
HPCN: Health post chief nurse
iCCM: Integrated community case management
LLIN: Long lasting insecticide treated net
NMCP: National Malaria Control Program
NGO: Non-governmental organization
ORS: Oral rehydration solution
PECADOM: Prise en charge a domicile
RDT: Rapid diagnostic test
RHMT: Regional health management team
SMC: Seasonal malaria chemoprevention
